# Regional Heterogeneity in 3D Myocardial Shortening in Hypertensive Left Ventricular Hypertrophy: A Cardiovascular CMR Tagging Substudy to the Life Study

**DOI:** 10.4236/jbise.2015.83021

**Published:** 2015-03-26

**Authors:** Robert W. W. Biederman, Alistair A. Young, Mark Doyle, Richard B. Devereux, Eduardo Kortright, Gilbert Perry, Jonathan N. Bella, Suzanne Oparil, David Calhoun, Gerald M. Pohost, Louis J. Dell’Italia

**Affiliations:** 1 Division of Cardiology, Department of Cardiovascular CMR, Gerald McGinnis Cardiovascular Institute, Allegheny General Hospital, Drexel University College of Medicine, Pittsburgh, USA; 2 University of Auckland, Auckland, New Zealand; 3 Weill Cornell Medical College, New York, USA; 4 ACTEK, Inc., Birmingham, USA; 5 University of Alabama at Birmingham, Birmingham, USA; 6 Albert Einstein College of Medicine, Bronx, USA; 7 University of Southern California, Los Angeles, USA

**Keywords:** Hypertension, Left Ventricular Hypertrophy, Magnetic Resonance Imaging, Cardiac Mechanics, Heart Wall Motion, 3D

## Abstract

**Background:**

Increased relative wall thickness in hypertensive left ventricular hypertrophy (LVH) has been shown by echocardiography to allow preserved shortening at the endocardium despite depressed LV midwall circumferential shortening (MWCS). Depressed MWCS is an adverse prognostic indicator, but whether this finding reflects reduced global or regional LV myocardial function, as assessed by three-dimensional (3D) myocardial strain, is unknown.

**Methods and Results:**

Cardiac Magnetic Resonance (CMR) tissue tagging permits direct evaluation of regional 3D intramyocardial strain, independent of LV geometry. We evaluated 21 hypertensive patients with electrocardiographic LVH in the LIFE study and 8 normal controls using 3D MR tagging and echocardiography. Patients had higher MR LV mass than normals (116 ± 40 versus 63 ± 6 g/m^2^, *P* = 0.002). Neither echocardiographic fractional shortening (32 ± 6 versus 33% ± 3%), LVEF (63% versus 64%) or mean end-systolic stress (175 ± 27 versus 146 ± 28 g/cm^2^) were significantly different, yet global MWCS was decreased by both echocardiography (13.4 ± 2.8 versus 18.2% ± 1.5%, *P* < 0.001) and MR (16.8 ± 3.6 versus 21.6% ± 3.0%, *P* < 0.005). 3D MR MWCS was lower at the base versus apex (*P* = 0.002) in LVH and greater in lateral and anterior regions versus septal and posterior regions (*P* < 0.001), contributing to the higher mean global MWCS by MR than echo. MR longitudinal strain was severely depressed in LVH patients (11.0 ± 3.3 versus 16.5% ± 2.5%, *P* < 0.001) and apical twist was increased (17.5 ± 4.3 versus 13.7 ± 3.7, *P* < 0.05). Importantly, both circumferential and longitudinal shortening correlated with LV relative wall thickness (R > 0.60, *P* = 0.001 for both).

**Conclusions:**

In patients with hypertensive LVH, despite normal LV function via echocardiography or CMR, CMR intramyocardial tagging show depressed global MWCS while 3D MR strain revealed marked underlying regional heterogeneity of LV dysfunction.

## 1. Introduction

Left ventricular hypertrophy (LVH) is a major adaptive response to chronic pressure overload and a powerful independent predictor of cardiovascular events in hypertensive patients [1]-[3]. However, measures of LV endocardial shortening, such as fractional shortening and ejection fraction, do not predict cardiovascular events in hypertensive LVH. In contrast, LV midwall circumferential shortening (MWCS), a measure of myocardial performance assessed by M-mode transthoracic echocardiography, has been shown to be decreased in a subset of patients with hypertensive LVH despite preserved LV ejection fraction [4]-[6]. Further, patients with LVH and decreased MWCS were at increased risk for cardiovascular events despite normal endocardial function [2]. In animal models of chronic pressure overload, functional studies of isolated papillary muscles demonstrated depressed contractility despite normal ejection fraction [8]-[10]. Thus, MWCS identifies a maladaptive hypertrophic response to chronic pressure overload. M-mode MWCS, however, is a global index of LV myocardial function derived from linear measurements of the anterior septum and inferolateral LV wall and therefore is unable to detect regional dysfunction of other myocardial segments [4]-[7]. Whether depressed MWCS represents globally uniform or regionally variable depression of LV myocardial function is unknown. Furthermore, it has been hypothesized that increased LV pressure could have dissimilar effects on wall stress in different LV regions due to differences in radii of curvature that could result in regional heterogeneity of load-induced myocardial dysfunction [11] [12]

Cardiac Magnetic Resonance imaging (CMR) radiofrequency (RF) tissue tagging allows intra-myocardial displacement and strain to be measured non-invasively by quantifying the motion of identifiable material points distributed throughout the myocardium [13]-[16]. Myocardial shortening in mildly hypertensive LVH patients has been suggested to be regionally heterogeneous by one-dimensional MR tagging [16] and more recently by us for two-dimensional MR tagging [17]. However, a full three-dimensional (3D) analysis has not been performed or compared with echocardiographic MWCS in LVH. These are necessary to ascertain whether depressed echocardiographic MWCS represents intrinsic global or regional dysfunction or effects of regionally heterogeneous afterload excess. Further, the identification of heterogeneous contractile performance in concentric LVH would point towards a pathophysiologic disturbance not predicted by the systemic processes that trigger hypertensive LVH, perhaps yielding insight into this significant clinical problem.

A complete description of 3D LV strain can be obtained by combining information from MR tagged short- and long-axis slices into a coherent 3D model of LV geometry and deformation [18]. This method has been validated using MR phantoms undergoing well-described deformations [19] and has been applied *in vivo* to normal volunteers and patients with hypertrophic cardiomyopathy [18]. Accordingly, we compared regional 3D LV deformation patterns from MR tagged images, as well as echocardiographic fractional and midwall shortening, in hypertensive patients with electrocardiographic (ECG) LVH enrolled in the Losartan Intervention for Endpoint reduction in hypertension (LIFE) study and in healthy volunteers. We hypothesized that 1) echocardiographic MWCS in well-compensated hypertensive patients with LVH reflects an averaged measure of global 3D LV function, 2) MWCS is heterogeneous in different LV regions, and 3) longitudinal as well as circumferential function is depressed, as assessed by 3D MR tissue tagging.

## 2. Methods

### 2.1. Patient Population

We studied 21 patients with essential hypertension and ECG LVH enrolled at our institution in the LIFE trial and the LIFE echocardiography substudy (Table 1) [20] [21]. Patients 55 - 80 years of age with blood pressure 160-200/95-115 mmHg were eligible for the LIFE study if their ECG demonstrated LVH (Cornell voltage-duration product > 2440 mm·msec and/or Sokolow-Lyon voltage SV_1_ + RV_5_ - V_6_ >38 mm) [20]. Exclusion criteria for this substudy included known LV ejection fraction < 50% or myocardial infarction within six months. Patients were studied at baseline after a 1-2-week supervised washout of antihypertensive agents, prior to beginning blinded medication. One patient had coronary angioplasty performed twice in the 5-year period prior to enrollment but had no evidence of active coronary artery disease and had an ejection fraction of 51% (echo). No other patients had prior evidence of coronary artery disease or alternative explanation for their LVH. Patients underwent a transthoracic echocardiogram and CMR tissue tagging examination within 24 hours. Four of the 21 patients imaged had MR studies that were unsuitable for complete 3D strain analysis, leaving 17 available for analysis. Eight normotensive volunteers (aged 25 - 38 years) with a normal ECG and no history of cardiac disease underwent the same echocardiographic and MR studies.

The protocol was approved by the Institutional Review Board for Human Use at the University of Alabama at Birmingham and all patients signed informed consent.

### 2.2. Echocardiography

Blood pressures were recorded by manual sphygmomanometer before and after the echocardiographic study. Two-dimensional (2D) and 2D targeted M-mode echocardiograms were recorded using a 2.25 MHz commercially available Hewlett Packard instrument. Linear measurements of interventricular septal and posterior wall thickness and LV chamber dimensions were measured from M-mode or 2D recordings according to recommendations of the American Society of Echocardiography [21]-[23]. All measurements were recorded in triplicate to minimize reproducibility error. LV mass was calculated by an anatomically validated method (r = 0.90, *P* < 0.001 versus necropsy measurements) [24].

MWCS was calculated according to the previously described method of de Simone and coworkers [2] [5]. The approach assumes a prolate spheroidal model, uniform contraction and wall thickening, and conservation of both inner and outer wall volume. This results in the following equation:

(1) $${\left({\mathrm{LVID}}_{d}+2h_{d}\right)}^{3}-{\mathrm{LVID}}_{d}^{3}={\left({\mathrm{LVID}}_{s}+2h_{s}\right)}^{3}-{\mathrm{LVID}}_{s}^{3}$$

where LVID is LV internal dimension, d is end-diastole, s is end-systole, and h is midwall thickness. Solving the above equation for h_s_ to yield the wall thickness at midwall at end-systole allows calculation of MWCS as

(2) $$\mathrm{MWCS}=100\%\times ({\mathrm{LVID}}_{d}+h_{d})-({\mathrm{LVID}}_{s}+h_{s})∕({\mathrm{LVID}}_{d}+h_{d})$$

End-systolic circumferential stress (σ_c_) was calculated according to the method previously described by Aurigemma and coworkers [4].

(3) $$\sigma_{c}=\frac{P(a^{2})(1+(b^{2}∕r^{2}))}{\left(b^{2}-a^{2}\right)}$$

where P is peak systolic pressure from cuff sphygmomanometer, a is endocardial radius, b is epicardial radius, and r is midwall radius.

### 2.3. MR Image Acquisition

**Figure 1:**
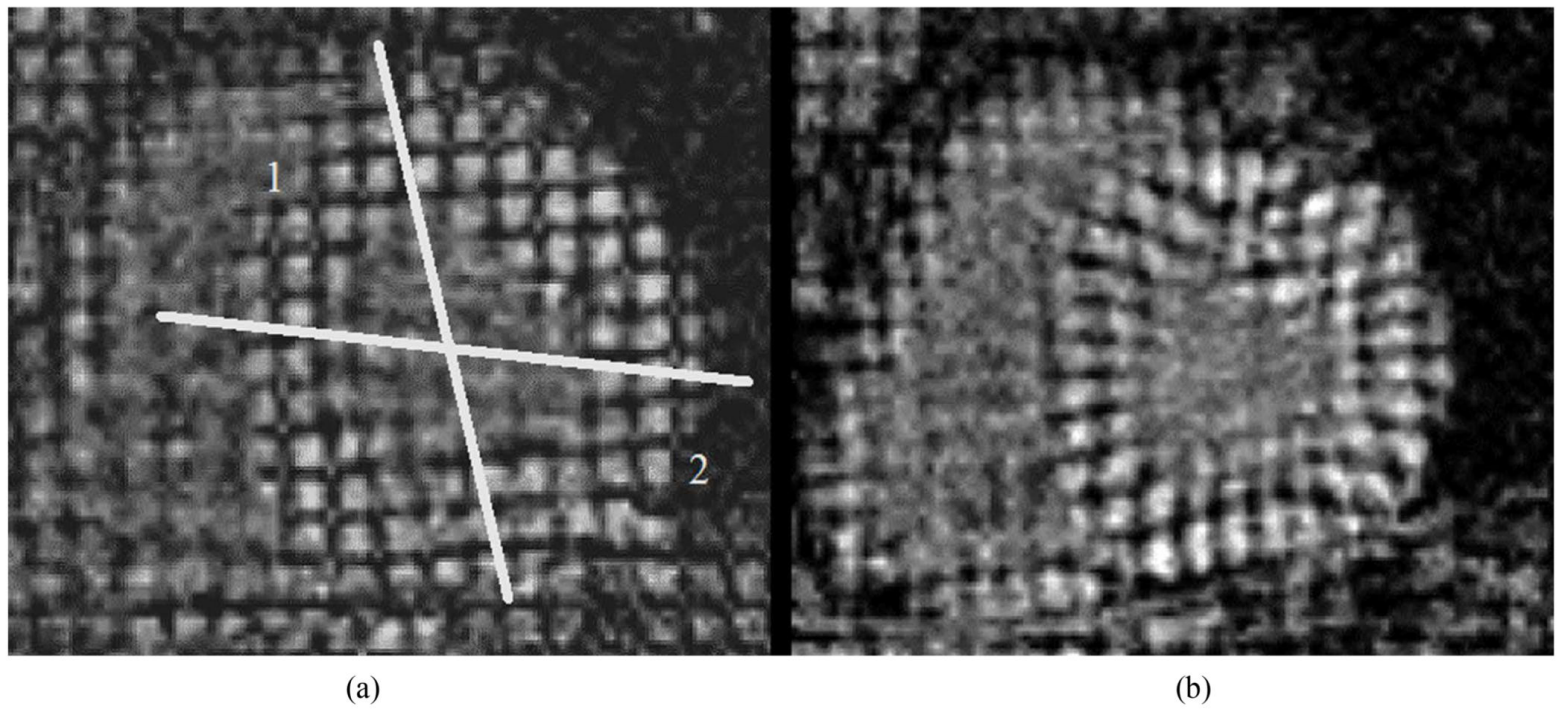
CMR short-axis tissue tagged images in a normal subject illustrating normal wall thickness and the high resolution tagging from end-diastole (a) to end-systole (b). Vertices of these tagging stripes were tracked through a cardiac cycle yielding strain information on a regional basis. Strain interrogation corresponding to those sampled by M-mode echo are shown. The anterior septum is Region 1 while the posterior lateral wall is Region 2.

**Figure 2:**
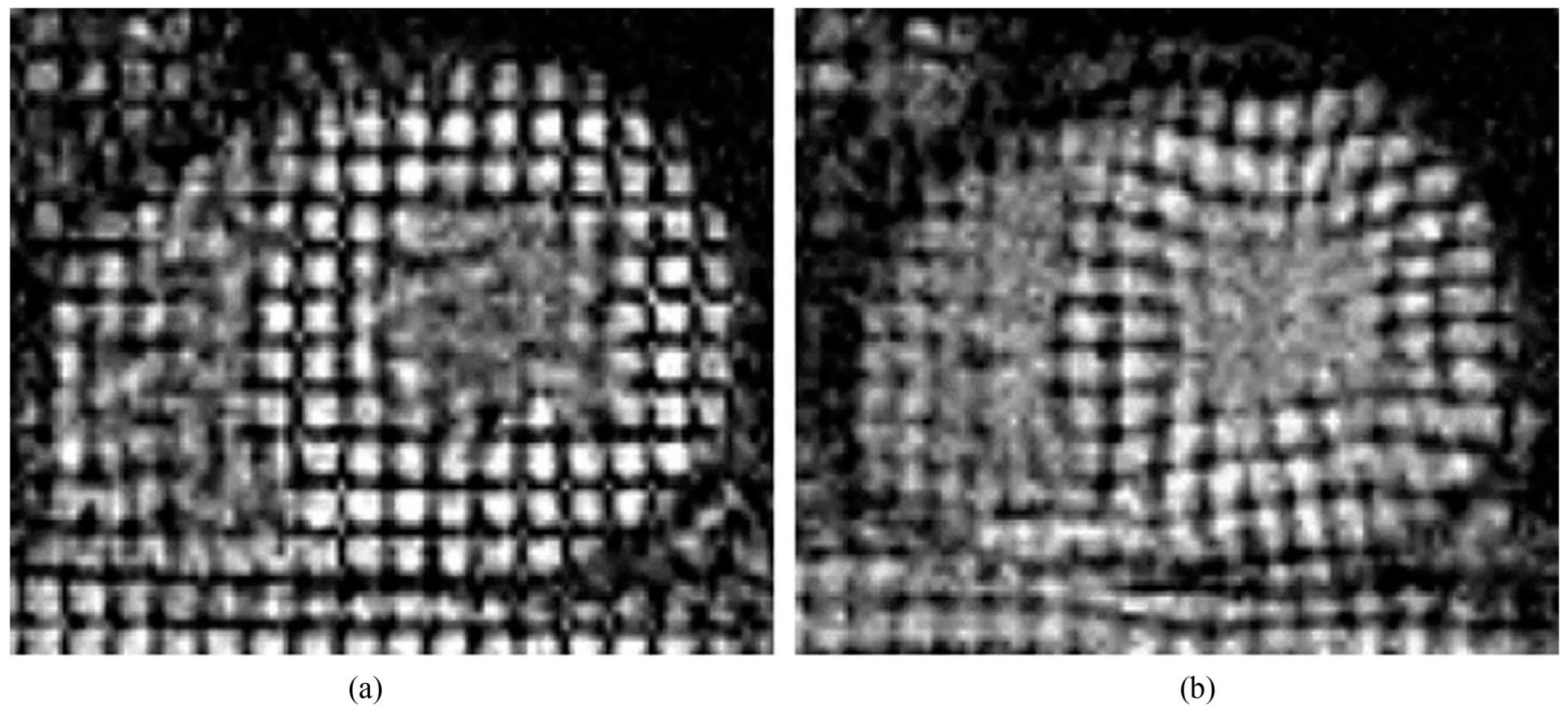
CMR short-axis tissue tagged images in a representative patient with hypertensive left ventricular hypertrophy illustrating increased wall thickness and high resolution tags. Vertices of these tagging stripes were tracked through a cardiac cycle yielding circumferential and radial strain information on a regional basis. Note how visual inspection alone reveals reduced septal deformation from end-diastole (a) to end-systole (b) compared with the normal subject in Figure 1.

Cardiac magnetic resonance imaging was performed with a 1.5 Tesla MR system (ACS Philips Best, The Netherlands). After performance of orthogonal scout scans, tagged images in the short axis, right anterior oblique, and four-chamber long-axis views were acquired with radiofrequency tags applied on detection of the ECG R wave. Three parallel slices were acquired for each of the two long-axis scans, with six slices acquired in the short-axis orientation. Scan parameters were TR/TE/flip 50/7.6/30, slice thickness 8 mm, interslice gap 4 - 10 mm, field of view 300 mm, matrix 256 × 256, and heart phase interval 50 ms. Orthogonal tags were produced by a five-element binomial pulse with composite flip angle of 160°, producing a tag spacing of 6 mm, using the previously described common k-space technique [20] [25]. To obtain adequate tag definition in the presence of unrestrained respiratory motion, a high degree of respiratory compensation was obtained using a modified respiratory ordered phase encoding (ROPE) compensation strategy taking input from a belt and bellows apparatus secured around the patient’s abdomen [21] [26]. The acquisition of k-space lines was synchronized with the respiratory cycle in a similar fashion to ROPE with the exception that for the respiratory segment with the highest excursion (*i.e*., during peak inhalation) data were not acquired. Both tagged and untagged short-axis cine images were acquired. Patients SBP was obtained for just prior to tag acquisition to measure simultaneous afterload and myocardial strain. Figure 1 and Figure 2 show representative examples of a normal control and patient, respectively. Figure 1 also demonstrates the CMR tag interrogation correlating with the echocardiographic parasternal acquisition for MWCS.

### 2.4. Image Analysis

Images were analyzed using custom-written interactive software running on an SGI O_2_ workstation. The inner and outer boundaries (endocardial and epicardial borders) of the LV were manually traced on the images so as to enclose the LV free wall and septum, excluding papillary muscles and trabeculae from the walls. The end-diastolic locations of the LV apex and the center of the LV at the base were defined on the most central long-axis slice. The intersections of the right ventricular endocardial boundaries with the septum were also defined at end-diastole in order to register each heart model to standard regions.

LV geometric models were fitted to the inner and outer contours of the untagged cine short-axis images, as described previously [27]. LV mass, end-diastolic and end-systolic volumes, ejection fraction, and stroke volume were calculated by numerical integration. Tag stripes within the muscle were located and tracked from end-diastole to end-systole using a previously described and validated tracking procedure based on an active contour model [14] [18]. To facilitate manual correction of the tracked stripe output, only the intersections between tag stripes were corrected and passed to the model-fitting procedure. The 3D locations of the final intersection points were calculated from the location of the image slice in space, which was encoded in the image header.

### 2.5. Finite Element Model

A 3D finite element model of the LV was constructed using previously reported methods [19]. The model interpolated the displacement constraints between tag and image planes, resulting in a consistent 3D displacement field. After the 3D displacements of all stripe points had been reconstructed, the model was used to estimate the deformation from end-diastole to end-systole by fitting the reconstructed displacements. The stripe tracking and model fitting procedures have been previously validated using a deformable phantom in the shape of a cylindrical annulus [19].

### 2.6. Motion Measurements and Statistical Analysis

Displacement and strain measures at any point in the model were calculated using standard methods of continuum mechanics [18] [19] [27]. Components of the Lagrangian strain tensor *E* were calculated with respect to the circumferential (C), longitudinal (L), and transmural (R) element coordinate directions. Components of *E* were used to calculate percent MWCS and midwall longitudinal shortening (MWLS) averaged over the middle third of the LV wall. LV twist was calculated as the rotation of the apex with respect to the base about the LV central axis. Midwall maximal principal shortening strain (MWPS) was calculated from the most negative eigenvalue of *E* and is associated with the maximum 3D contraction experienced by the tissue at each material point [19].

### 2.7. Statistical Analysis

Displacement and strain data were averaged in 17 standardized regions in accord with recommendations of the American Heart Association [22], with region 17 (apical tip) ignored due to partial voluming [23]. The LV was divided into three longitudinal portions (apex, mid, and base), which in turn were divided circumferentially into four (at the apex) or six (at mid-ventricle and base) regions. Repeated measures ANOVA were used to test for regional differences in displacement and strain, and differences in global midwall shortening (MWS) using MR and echocardiography. Mean LV mass, echocardiographic MWS, circumferential end-systolic stress, and shortening and ejection fraction were compared between normals and patients by Student’s unpaired *t* tests. Two-tailed *P* < 0.05 was required to achieve statistical significance.

### 2.8. Author Statement

The authors had full access to the data and take responsibility for its integrity. All authors have read and approve the manuscript as written.

## 3. Results

### 3.1. Echocardiography

Echocardiographic and blood pressure data are shown in Table 1. Mean end-diastolic septal and posterior wall thicknesses were greater in hypertensive patients than in normal adults, with minimally larger LV internal dimensions. As a consequence, LV mass was two-fold greater in patients versus normals and LV relative wall thickness was substantially higher in the former group. All patients had septal: posterior wall ratios ≤ 1.05:1 excluding occult phenotypic hypertrophic cardiomyopathy. Neither LV fractional shortening nor ejection fraction differed between groups. End-systolic circumferential wall stress was slightly but non-significantly higher in hypertensive patients, who had significantly lower echocardiographic MWCS. However, MWCS did not strongly correlate with LV mass, LV mass index, or systolic blood pressure in hypertensive patients.

### 3.2. CMR

Table 2 shows global functional data obtained from the CMR images. LV mass was significantly higher in the LVH group, as was LV mass/body surface area. Mean LV mass by CMR was >2-fold greater in LVH patients versus normal adults and >3 SD’s above normal. While echocardiographic end-diastolic and end-systolic dimension were similar between groups, 3D CMR end-diastolic and end-systolic volume were slightly decreased in patients compared with the control group. Independent quantification of LVEF by Echo and CMR confirmed nearly superimposable LV function (>60% for all via CMR).

Tags were reconstructed to end-systole in all cases. The root-mean-square error between the reconstructed material point locations from the fitted model and the corresponding tracked stripe data points averaged 1.0 mm for the control group, and 1.2 mm for the LVH group at end-systole. These were comparable to the pixel size of 1.17 mm. Circumferential shortening averaged over the midwall third of the LV model (MWCS) was reduced in the LVH group (Table 2). Average longitudinal shortening in the midwall (MWLS) was severely depressed and 3D midwall principal contraction strain (MWPS in Table 2) was mildly reduced in the patient group. Average LV twist (relative rotation of the apex with respect to the base) was increased in the patient group (Table 2).

**Figure 3:**
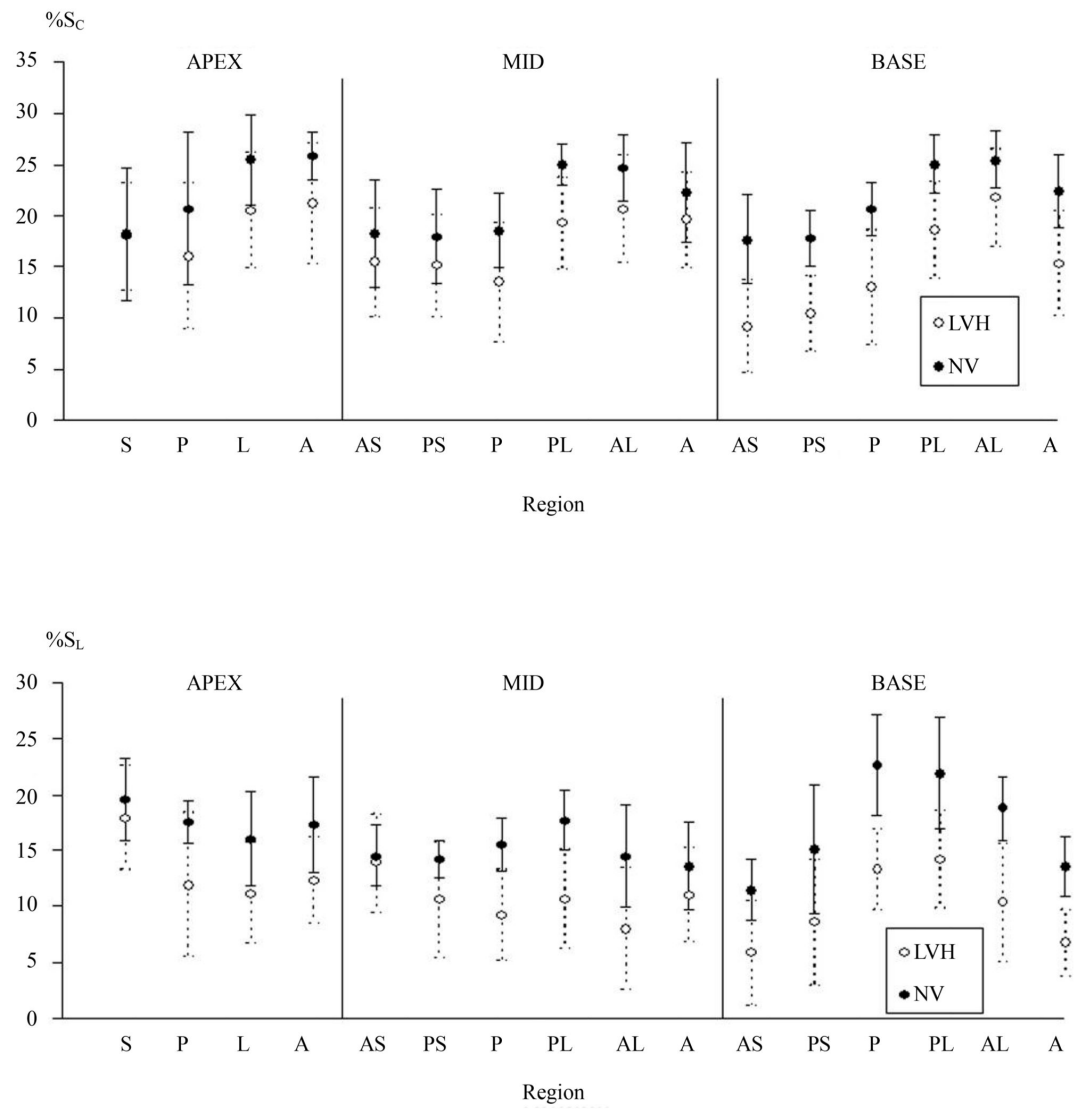
Percent circumferential (S_C_) and longitudinal (SL) strain in patients with left ventricular hypertrophy (LVH) and normal volunteers (NV) demonstrating heterogeneity of regional intramyocardial performance as a function of level in the ventricle and ventricular wall. Myocardial function by MR tissue tagging is depressed in LVH patients as compared with normal volunteers to variable degrees in different myocardial segments. The gradient in dysfunction is more apparent at more basal ventricular levels.

**Figure 4:**
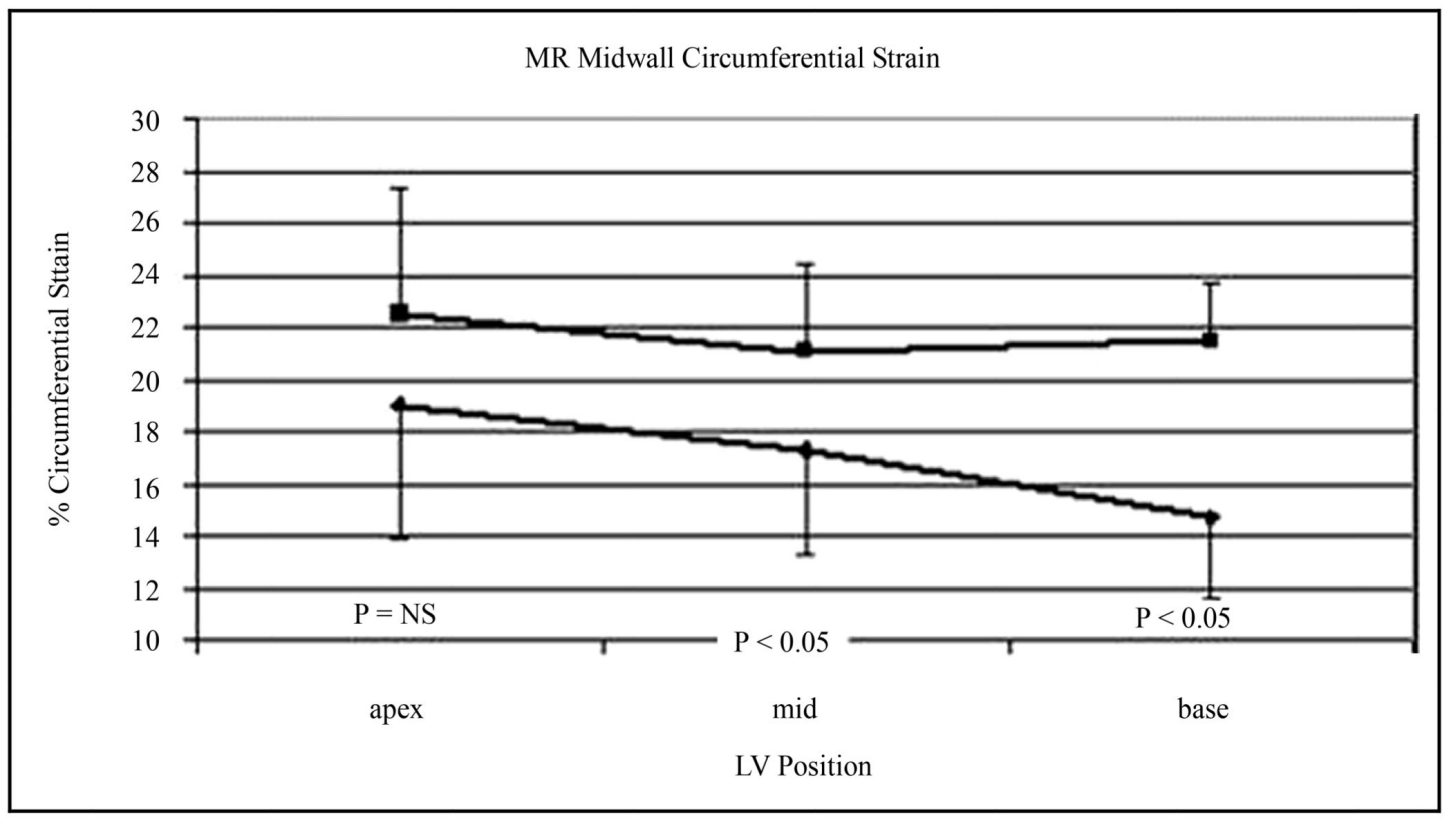
3D circumferential strain is uniform throughout the non-hypertrophied ventricle at apex, mid-ventricle and basal LV levels (upper line). In comparison, hypertrophied ventricle is distinctly heterogeneous in its midwall circumferential strain: a longitudinal gradient, with decreasing circumferential strain from apex to base is evident (lower line). (ANOVA comparison for circumferential strain between levels in hypertensive LVH patients *P* < 0.05 for base and mid-ventricle compared with the apex.)

**Figure 5:**
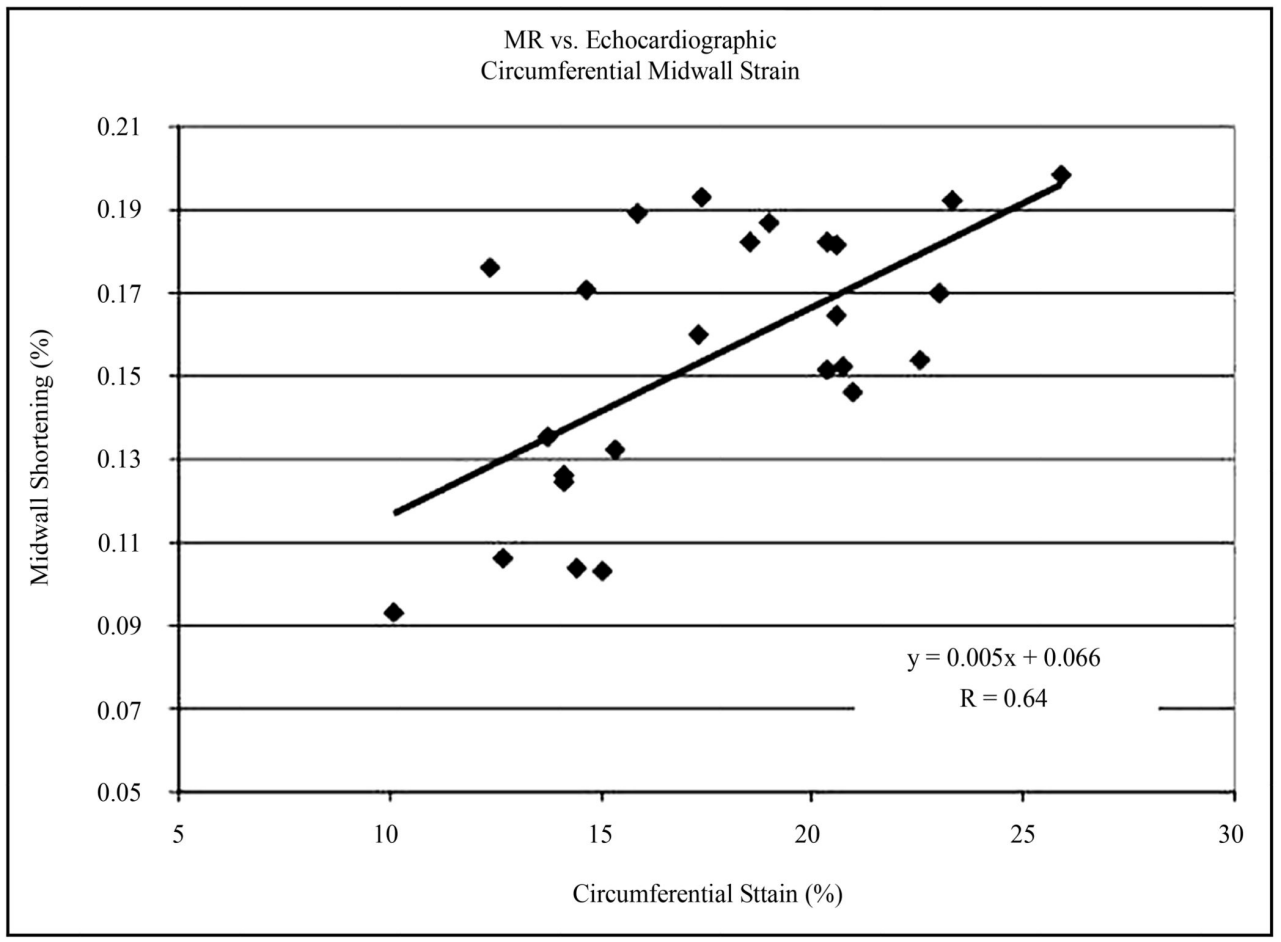
LV midwall mechanics by MR or echocardiography: 3D MR circumferential strain versus M-mode midwall shortening (MWS). Comparing classic 2D approach for echocardiographically determined MWS against MR-derived 3D midwall circumferential strain, there is a high degree of correlation between, despite significant regional non-uniformity.

Regional variation in MWCS and MWLS are shown in Figure 3. In LVH patients and normal volunteers, there was significant regional variation in MWCS and MWLS (ANOVA *P* < 0.001 for both, Figure 3). MWCS was greater in lateral and anterior walls than septal and posterior walls in both groups (*P* < 0.001), whereas MWLS was greater in posterior and lateral walls than in the septum (*P* < 0.001). When divided into apical, midventricular, and basal thirds, MWCS and MWLS were greater in the apex than the base (*P* = 0.002 and *P* < 0.001, respectively) in the LVH group (Figure 4). Differences between patient and normal groups were also regionally heterogeneous (*P* = 0.005). Relative to the normal group, patients’ MWCS was statistically similar at the apex (19.0 ± 5.0 versus 22.5% ± 4.8%, *P* = NS), moderately reduced at midventricle (17.3 ± 4.1 versus 21.1% ± 3.4%, *P* = 0.02), and greatly reduced at the base (14.8 ± 3.2 versus 21.5% ± 2.1%, *P* < 0.001), supporting a longitudinal gradient in circumferential myocardial function in hypertensive LVH that was not present in normal LV’s (Figure 4). In contrast, MWLS was significantly reduced at the apex, midventricle, and base (*P* < 0.02 for each) compared with normals. Importantly, the two regions used for echocardiographic calculation of MWCS from linear dimensions had significantly different MWCS and MWLS by CMR tagging, with 102% greater circumferential and 140% greater longitudinal shortening in the posterior lateral wall than in the anterior septum in the LVH group (*P* < 0.01 for each) (Figure 3). At the base, the agreement between average echocardiographic and MR MWCS was good, varying less then 15% (13.4 ± 2.8 versus 14.8% ± 3.2% respectively for patients and 18.2 ± 1.5 versus 21.5% ± 2.1% for normals, Figure 5).

MWCS and MWLS were negatively related to wall thickness to radius ratio (RWT) (r = −0.60 and r = −0.70, respectively, both *P* < 0.001), with increasing RWT associated with decreasing shortening. Controlling for RWT as a covariate, there was no difference in midwall shortening between normals and LVH patients (ANOVA *P* = NS), indicating that degree of concentric hypertrophy appears to partially explain the lower shortening in LVH patients than in normal adults.

## 4. Discussion

Hypertensive LVH patients with subnormal overall MWCS by echocardiography despite normal LV chamber function have increased morbidity and mortality [2]. We have recently identified regional heterogeneity of LV myocardial function in hypertensive LVH by 2D MR interrogation [17], but to the best of our knowledge, a complete analysis incorporating assessment of x, y and z axis strain has not been reported [17]. To assess the 3D contraction patterns in these patients, we tracked material points in the form of magnetic tags through systole in short- and long-axis MR image series to reconstruct 3D LV motion. MWS in circumferential and longitudinal directions was found to be regionally heterogeneous, with reduced shortening at the base relative to the apex and midventricle, and significant circumferential variation within each level. In particular, the two regions used to derive the echocardiographic MWCS have significantly disparate shortening, with more normal shortening in the posterolateral LV wall than the anterior septum at the base. Despite this regional heterogeneity, echocardiographic MWCS was decreased in LVH patients to a similar extent to MWCS estimated from 3D MR tagging. Mean global MWCS was higher by MR tagging than by echo in patients and controls, in part because shortening in apical and midventricular regions was higher than basal shortening, and lateral shortening was higher than septal shortening. Importantly, the agreement between average echocardiographic and MR MWCS was good, varying <2% in absolute terms.

It is important to note that 3D intramyocardial properties represent near total expression of LV strain, yet 2D strain analysis is more commonly used both clinically and experimentally. As such, echocardiographic-derived MWS deserves comparison with 2D-derived MR calculation of midwall performance. Therefore, 2D strain was calculated and found to be similar to echocardiographic MWS in patients and controls (13.4 ± 2.8 versus 13.9% ± 6.8% (septum) and 18.2 ± 1.4 versus 22.4% ± 3.5% (posterior wall), *P* = NS for both) [17].

Heretofore it was unknown whether decreased echocardiographic MWCS in patients with hypertensive LVH represented intrinsic myocardial dysfunction or was related to geometric assumptions inherent in the calculations, thereby introducing possibly artifactual depression due to geometric effects of LVH. For example, based on the formula used to calculate echocardiographic MWCS, any distance traveled by the midwall will be inherently reduced by a greater initial diastolic wall thickness [5]. Indeed, MWCS has been shown to correlate negatively with LV mass and relative wall thickness in large population studies [2]. Moreover, echocardiographic MWCS is designed to represent a global index under the assumption that myocardial function in hypertensive LVH is homogeneous and symmetrical. Our observations using CMR techniques suggest that observed MWCS seen by echocardiography tracks intrinsic myocardial dysfunction. However, this reflects contributions of markedly different posterior wall and basal septal contractions that do not capture full heterogeneity of LV myocardial shortening. Moreover, in patients with preserved chamber function, normal end-systolic stress, and similar posterior wall and septal thicknesses, depressed echocardiographic MWCS would not predict an asymmetrical contractile pattern.

In a previous study of hypertensive LVH patients with normal ejection fraction using 1D MR tagging, Palmon *et al.* [16] showed reduced MWCS and MWLS compared with normal subjects. A similar pattern of regional heterogeneity of circumferential shortening was observed in the present study, with greater shortening in the lateral wall than the inferior wall. However, average values of MWCS were higher than the present study (20% ± 6% versus 17% ± 4% for the present study). One reason for this may be that the 1D analysis employed in the previous study could not track material points, unlike the 3D analysis of the present study. Additionally, there was ECG evidence of LVH and a substantially greater echocardiographic LV mass in our group (163 ± 52 versus 127 ± 37 g/m^2^) [23].

Geometric effects due to concentric LV hypertrophy characterizing these patients may explain reduction in MWCS and MWLS observed in the patient group. Cylindrical models of LV function indicate that increasing RWT (wall thickness/chamber radius) allows less midwall myocardial shortening for the same ejection fraction. In a study of regional 3D myocardial shortening patterns in patients with hypertrophic cardiomyopathy [18], similar reduction of circumferential shortening and a similar pattern of regional heterogeneity as in the present study were observed. Longitudinal shortening was severely depressed in both the present study and in patients with hypertrophic cardiomyopathy [18]. However, LV twist and ventricular torsion were increased, possibly due to increased lever arm of the epicardial fibers. Explanations for decreased strain in setting of preserved global systolic function in patients with concentric LVH have been confirmed recently by us via CMR in aortic stenosis [28] and most recently in a separate cohort of hypertensive LVH [29].

The explanation for these observations in hypertensive LVH may be related to the distinct septal architecture in which separate raphe fibers from the right ventricle insert into the septum but are distinctly absent from the posterior wall. Physiologically, MWCS (echo and CMR) and MWLS (CMR) may be abnormal in LVH due to a geometric consideration. Namely reduced endocardial excursion is required when there is sarcomere hypertrophy in parallel, a feature detectable by echocardiography but one not expected to be asymmetric by either technique [30]. Whether reduced septal strain is a harbinger of clinical events is currently a focus of our ongoing work [29].

### 4.1. Limitations

One possible limitation of this study is that the LVH patients were older than the normal volunteers (56 - 78 versus 25 - 38 years). However, Slotwiner and coworkers [31] demonstrated in 464 patients that echocardiographic MWCS did not differ between normal subjects aged 16 - 41, 41 - 54, and 54 - 88 years. A similar observation was made by Palmon *et al.* [16]. This argues against non-uniformity of myocardial contraction being an age-related phenomenon. In a study of effects of aging on CMR-derived regional strain parameters, Oxenham *et al.* [32] compared 15 normal volunteers 19 - 26 years old with 16 normal volunteers 60 - 74 years old. Similarly, Augustine *et al* recently showed little impact of gender based strain differences [33]. Finally, in the pediatric population, strains appear similar [34] suggesting this is not an age-related phenomenon.

Systolic circumferential and longitudinal shortening were statistically similar in the two groups, whereas apical rotation and ventricular torsion were mildly increased. In contrast, diastolic relaxation of circumferential and longitudinal strain was severely depressed in older versus younger normals. Unfortunately, we were unable to track tags beyond end-systole in the present study, but this will be possible with improved scanning hardware and software.

Given the reproducibility of CMR, the number of patients needed to be examined is far less then echocardiography. This concept has been well recognized [35]. Further support is demonstrated in our power calculations demonstrating a power of 0.84 (p < 0.05) for reduced MWCS and MWLS as compared to normal subjects.

Late gadolinium enhancement (LGE) was not performed by design in this study. As such, future integration of LGE with deformation analysis might yield even further insights into the pathophysiology of this observation.

### 4.2. Conclusion

Despite the use of highly simplified assumptions, echocardiographic MWCS was depressed to a similar extent to direct 3D MR tagging estimates of MWCS in hypertensive LVH patients with normal chamber function and end-systolic stress. In the basal third of the LV where echocardiographic MWCS is obtained, the two estimates of MWCS were similar; however, a gradient in shortening was present increasing towards the apex in ventricles with hypertensive LVH. Regionally, 3D CMR demonstrated marked heterogeneity in intramyocardial dysfunction undetectable by standard echocardiographic indices. Thus, the observed decrement in echocardiographic MWS is a function of regional dysfunction that parallels but does not precisely reflect global dysfunction. The presence of concentric hypertrophy plays a major role in the depression of MWS; however, the shortening pattern exhibits marked regional heterogeneity, leading to an underestimation of global MWCS by echocardiography. Heterogeneous contractile performance detectable by 3D CMR in the otherwise symmetrical process of concentric LVH suggests that 3D analysis of LV midwall function in larger patient series may help identify underlying LV mechanical dysfunction in even more hypertensive patients with heart failure than is detected by conventional echocardiographic analyses [30].

### 4.3. Clinical Perspective

Cardiac CMR permits interrogation of cardiac mechanics with fidelity unparalleled by traditional non-invasive or invasive modalities. For the first time a complete 3D examination of the common clinical entity of hypertensive LVH has been performed identifying myocardial *heterogeneity* in a classically presumed *homgeneous* hypertensive pathophysiological process. This new insight now confirms that the heretofore presumed homogeneous contractile pattern in concentric hypertrophy should now be reconsidered to demonstrate heterogeneous regional myocardial deformational variations and perturbations not visible by lower-resolved imaging strategies.

## Figures and Tables

**Table 1:** Clinical and echocardiographic characteristics.

	Patients	Normals

Sex	12 Males, 5 Females	7 Males, 1 Female
Body surface area	2.26 ± 0.23	2.17 ± 0.21
Blood pressure, mmHg	178/94 ± 14/14*	119/70 ± 9/5*
Heart rate	71 ± 11	75 ± 7
Posterior wall thickness diastole, cm	1.24 ± 0.22*	0.75 ± 0.08*
Septal wall thickness diastole, cm	1.29 ± 0.24*	0.79 ± 0.06*
LV end-diastolic dimension, cm	5.21 ± 0.36	5.00 ± 0.44
LV end-systolic dimension, cm	3.63 ± 0.35	3.34 ± 0.37
Relative wall thickness	0.48 ± 0.08*	0.30 ± 0.03*
LV mass, g/m^2.7^ (echocardiographic; allometric)	65.7 ± 23.4*	28.1 ± 6.0*
Septal/posterior wall ratio	1.04:1	1.05:1
End-systolic circumferential stress, g/cm^2^	175 ± 27	146 ± 28
Fractional shortening, %	32 ± 6	33 ± 3
Ejection fraction, %	64 ± 11	65 ± 5
Echo MWS, %	13.4 ± 28^†^	18.2 ± 1.5^†^

LV indicates left ventricular; LV mass 2.7, LV mass normalized to the allometric power of height (2.7); MWS, midwall shortening.

* *P* < 0.005 patients versus normals;

† *P* < 0.05 patients versus normals.

**Table 2:** Cardiac magnetic resonance findings.

	Patients	Normals
Mass by CMR, g	220.4 ± 73.7*	121.0 ± 24.2*
Mass by CMR normalized by BSA (g/m^2^)	97.5 ± 32.6*	55.7 ± 11.2*
End-diastolic volume, ml	109 ± 24^†^	131 ± 25^†^
End-systolic volume, ml	40 ± 17^†^	47 ± 14^†^
LVEF, %	63.3 ± 2.9	64.1 ± 3.1
MR midwall circumferential strain, %	16.8 ± 3.6*	21.6 ± 3.0*
MR midwall longitudinal strain, %	11.0 ± 3.3^†^	16.5 ± 2.5^†^
Maximum principal strain, %	23.8 ± 4.0*	27.6 ± 2.2*
Twist (torsion)	17.5 ± 4.3*	13.7 ± 3.7*

BSA indicates body surface area; CMR, magnetic resonance imaging.

* *P* < 0.05 patients versus normals;

† *P* < 0.001 patients versus normals.
